# Factors influencing planning of a familiar grasp to an object: what it is to pick a cup

**DOI:** 10.1007/s00221-017-4883-x

**Published:** 2017-02-16

**Authors:** Elisabeth Rounis, Zuo Zhang, Gloria Pizzamiglio, Mihaela Duta, Glyn Humphreys

**Affiliations:** 1 Nuffield Department of Clinical Neurosciences, University of Oxford, Level 3 West Wing, John Radcliffe Hospital, Oxford, OX3 9DU UK; 2 Department of Experimental Psychology, University of Oxford, Oxford, UK; 3 Department of Computer Science and Technology, Tongji University, Shanghai, China

**Keywords:** Affordances, Motor preparation, End state comfort, Reaction times

## Abstract

We assessed the factors influencing the planning of actions required to manipulate one of two everyday objects with matching dimensions but openings at opposite ends: a cup and a vase. We found that, for cups, measures of movement preparation to reach and grasp the object were influenced by whether the grasp was made to the functional part of the object (wide opening) and whether the action would end in a supinated as opposed to a pronated grasp. These factors interacted such that effects of hand posture were found only when a less familiar grasp was made to the non-functional part of the cup (the base). These effects were not found with the vase, which has a less familiar location for grasping. We interpret the results in terms of a parallel model of action selection, modulated by both the familiarity of the grasp to a part of the object, likely to reflect object ‘affordances’ and the end state comfort of the action.

**Electronic supplementary material:**

The online version of this article (doi:10.1007/s00221-017-4883-x) contains supplementary material, which is available to authorized users.

## Introduction

When planning to grasp an object, we are faced with an infinite number of combinations of arm postures and movements which could be used to achieve a particular goal (Bernstein 1967; Wolpert 1997). However, humans engage in highly stereotyped movements to achieve a given action (Keele 1968; Harris and Wolpert 1998). Studies have shown that action plans are organised around temporally distal outcomes or goals (Rosenbaum et al. 2006; Grafton and Hamilton 2007). When manipulating objects, grasp movements may be influenced by object shape, position and size (i.e. ‘perceptual’ factors—Jeannerod 1994), as well as by what one intends to do with the object (Marteniuk et al. 1987; Rosenbaum et al. 2006).

One example of the latter is the end-state comfort effect originally described by Rosenbaum et al. (1990, 1992). This reflects the observation that participants often select grip postures that may be uncomfortable at the start of an action to end in a comfortable posture (which might enable object use). The end-state comfort effect is thought to reflect a constraint on the choice of one action from an overwhelming number of possible others when planning to reach and grasp an object (Miall and Wolpert 1996), removing the need to programme new movement trajectories each time (Wong et al. 2015).

Rosenbaum’s original description of an end-state comfort effect was based on the observation made at a restaurant, of a waiter choosing to pick cups from a table that were inverted with a pronator grasp at the start, to end with a supinated grasp allowing them to be filled (Rosenbaum et al. 2006). However, original studies investigating the end-state comfort effect have involved selection of hand postures to grasp an abstract, rather than a familiar, object. In their first experiment, participants were asked to grasp a horizontal bar to transport it from its centre cradle to either a left or a right disc in a vertical position (Rosenbaum et al. 1990). The task required a particular side of the bar to be placed on the disc but participants were allowed to choose which grip to use to achieve this (i.e. either an overhand or underhand grip). The authors observed that participants chose to grasp the bar in a way that resulted in a comfortable hand posture at the end of the task. Similar observations were made in later studies [e.g. the ‘grasp height effect’ in which people grasp an object near its bottom when moving it higher, Rosenbaum et al. (1990, 2014)].

In addition to observing an effect on the choice of grasp to move an object, one study focused on how end-state comfort effects emerged over time, using measures of reaction time (RT) and movement time (MT) (Rosenbaum et al. 1992). In that study, participants had to reach out and move a bar from one of four possible locations from a central display to one of eight target locations displayed in the periphery. A piece of tape wrapped around one end of the bar served as a pointer, whilst light-emitting diodes in the periphery served as targets; indicating an orientation in the object being used. Participants chose their grasp postures in a manner that was consistent with the end-state comfort effect. In addition, there were differential effects of perceptual and end-state comfort effects on the participants’ reaction and movement times (RTs and MTs). The former appeared to be influenced by the starting thumb orientation in the grasp: RTs were shorted when participants grabbed the bar with the thumb toward the pointer rather than when it was away. However, this ‘thumb toward’ effect depended on the target location, and was reversed (into a ‘thumb away’ preference), when one of the eight locations led to an awkward movement. The MTs demonstrated an influence of the end-state comfort effect. MTs differed for identical start and target positioning of the bar, depending on the participants’ choice of grasp: they were shorter for grasps that ended comfortably.

It remains unclear how such planning effects may interact with properties of a known object (e.g. its size, shape, or orientation) or the environment, which are also likely to influence action (Gibson 1979; Jeannerod 1994). Studies examining perceptual effects of object properties on hand actions (referred to as ‘affordances’) describe stimulus–response compatibility effects based on a correspondence between the graspable features of an object and an independent action that has to be elicited in the task. These studies have used response times to measure these effects, with shortening of these times corresponding to ‘compatible’ trials. For example, Tucker and Ellis (1998) instructed participants to make finger presses with their right- or left-hand, according to whether objects in pictures were depicted as upright or inverted. They found that reaction times (RTs) were shorter if the orientation of the handle of the object with respect to the hand used for the response was compatible, even though participants were not required to make a judgement about the handle orientation itself. The question arises as to whether the cognitive representation of the object influence the possible action representations to it (Ellis and Tucker 2000; Tipper et al. 2006; Cisek 2007).

Most of the studies described above, have looked at the perceptual effects of familiar object-oriented actions and the effects of planning using end-state comfort, separately. Surprisingly, this issue, of the dynamic inter-play between the two, has not been examined. For example, a highly familiar action to an object may counter any effects of forward planning (and end-state comfort), even if the object subsequently has to be placed in an orientation with an uncomfortable end-state action.

We developed a new experimental procedure where we varied the compatibility of the initial start posture of the hand with the physical properties of a target object, and the end posture for the action (whether it ended comfortably or not). We were interested in measuring how these effects interacted during motor planning. To this end, we used measures of participants’ reaction and movement times (Henry 1952; Welford 1968). Participants’ reaction time (which we termed their ‘initiation time’) measured the time of initiation of a reach-to grasp movement to the object. This was marked by the release of their hand from a resting position they were instructed to press at baseline. Their ‘movement duration’ measured the time between the release time and the time the object was lifted from a response platform to perform the action that was instructed upon it. Whereas the former, initiation time, has been described as representing the time when a decision of what action to implement takes place, and possibly how to implement it (at an abstract level) (Wong et al. 2015); the movement duration is more likely to describe processes relating to ‘how’ to implement it, whilst reaching to grasp the object. Of note, there is recent literature in which object congruency effects have been shown the latter timings (Bub and Masson 2010; Wilf et al. 2013). We propose a broader definition of motor planning in this study, to incorporate planning components identified in the initial, reach-to-grasp, part of movement execution (Bub and Masson 2010; Wilf et al. 2013). This is further to neurophysiology evidence indicating that some components of planning continue during the execution phase of the response (Cisek 2005).

Based on previous literature, we predicted we would observe one of two possible patterns of responses, relating to our effects of interest, using these timings. One possibility was that perceptual features of the object would be present early on, and indexed in the initiation times, whereas effects relating to the end-state comfort would be most prominent during action implementation, indexed by the movement durations (as suggested in the study by Rosenbaum et al. 1992). Another possibility predicted the opposite pattern of responses: indeed end-state comfort might be present early on in movement planning, to indicate its predictive effect on action responses (Zimmermann et al. 2016), whereas effects of compatibility of the initial hand grasp with the object orientation, would emerge over time, as the hand approached the cup. This has been suggested by recent studies in the literature, which argue that these hand-object compatibility effect may emerge over time (Bub and Masson 2010) and that compatibility effects, such as the Simon effect, can be sustained, particularly if a response involves a more complex mapping between spatial representation and action (Buhlmann et al. 2007; Bub and Masson 2010).

In our study, participants were instructed to either lift or turn a cup presented in front of them by grasping it from a specified position (namely its top, which corresponded to the open end of the object that could be filled, or its bottom, which corresponded to the closed end). The cup itself was placed in an upright orientation in one-half of the trials or upside down in the other half. This led to four possible actions: two lift actions (lift with a supinated grasp or lift with a pronated grasp) and two turn actions (turn with a supinated grasp, ending in a pronated (uncomfortable) posture; or turn with a pronated grasp, ending with a supinated (comfortable) posture).

Our first hypothesis was that we would identify effects of (1) an initial grasp preference, which was most likely related to the physical properties and familiarity of the grasp to the object, and (2) an end-state comfort effect, related to whether the posture of the hand at the end of the task was comfortable or not. This is based on literature identifying separable neural networks involved in these processes, within the dorsal and ventral streams (Grezes et al. 2003; Mruczek et al. 2013; Zimmermann et al. 2012, 2016).

**Fig. 1:**
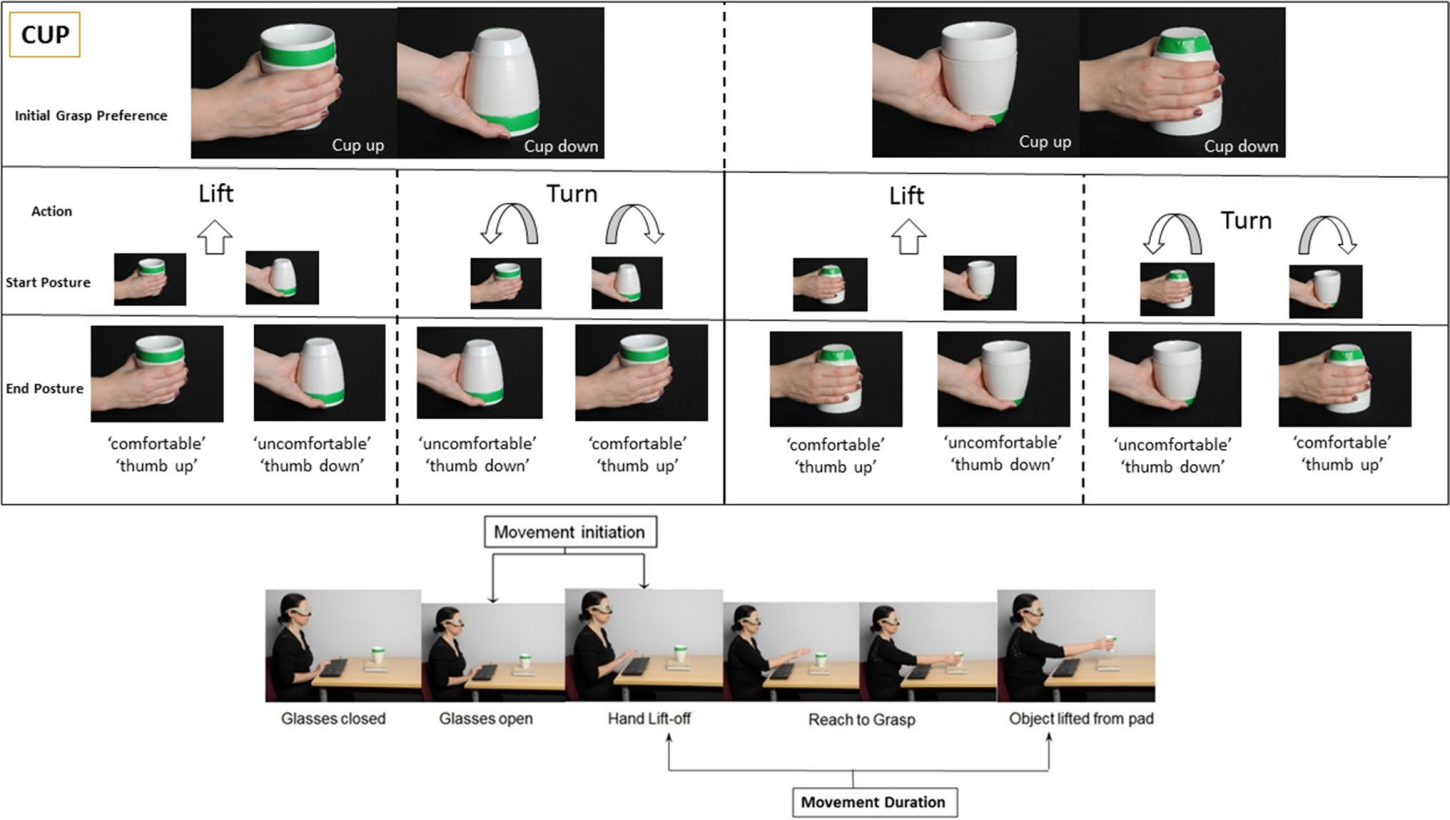
*Top panel* shows the eight task conditions. We manipulated the action participants had to make on the object (to ‘lift’ or to ‘turn’ it), the initial hand position to grasp the object, which was demarcated by a *green line* positioned either to the *top* or to the *bottom* of the cup: this was either aligned to the open end of the cup (wider side), meaning that the cup was being grasped with a familiar grasp, from its open end, and the end state comfort which depended on the action and the initial hand posture, and was either comfortable (in a thumb *up* position) or uncomfortable (ending with the thumb ‘*down*’). Four actions specified by the task conditions: lift with supinated grasp, lift with pronated grasp, turn with supinated grasp, turn with pronated grasp; the *bottom panel* shows an example trial (to lift the cup, which was oriented upright, with their hand positioned at the *top*, wider end, i.e. congruent with the cup orientation). The two times measured in this experiment included an initiation time representing the time to liftoff the hand from a keyboard to initiate the action, and movement duration which represented the time to reach and grasp the object (between liftoff of the hand from its resting position to lifting the object from the response pad). (Color figure online)

In a second experiment, we aimed to confirm results from the first experiment investigating the initial grasp effects by exploring whether this preference related specifically to the physical properties of the object used. As the initial grasp preference for the cup was to the wider end of the object, rather than its narrow end, could this preference have arisen from the fact that grasping the wide part of the object was felt to be ‘easier’ than grasping the narrow part or was this because the wide end of the object was also the open (functional) end? A new group of participants were tested on a modified object, namely a conical-shaped vase, made from the same object (the cup) thus with identical physical properties but with the open end on the opposite side (Fig. 1). The aim was to investigate whether any difference in performance identified with the former object (the cup) related to physical properties of the object or its function.

## Experiment 1: reaching and grasping a cup

### Methods

#### Participants

Thirty healthy participants [mean age was 25.8 years, *M* = 14:*F* = 16] were recruited from advertisements in the Department of Experimental Psychology. All participants were right-handed (Oldfield 1971), had normal or corrected-to-normal vision, and provided informed consent in accordance with the University of Oxford ethics committee. The experiment took approximately 1 h to complete and participants were reimbursed for their time and travel.

#### Materials

A cup, which was shaped as an inverted cone, with no handle (‘bodum’ cup—Fig. 1) was used as the target object in this experiment. This choice of object, namely a cup with no handle, was aimed to prevent lateralizing effects of visuospatial attention (Rushworth et al. 2001). The cup dimensions were: 50 mm wide at its base, 98 mm wide at its top, and 118 mm height, and it weighed 270 g. The centre of mass of the cup was located 7.6 cm from its base. It was placed at the centre of a wooden platform measuring 20 cm-by-10 cm that sat on top of a cedrus response box 30 cm in front of the participants. On half the trials the cup was upright and in the remaining half it was oriented upside down.

#### Task

Participants used their dominant (right) hand to perform this task. Their hand rested on a keyboard at baseline, between trials. A trial started with the opening of liquid crystal ‘PLATO’ spectacles (Translucent Technologies, Toronto, Ontario, Canada). This allowed the timely visualisation of the object on the pad. A simultaneous verbal auditory instruction triggered from the computer, lasting 1 s, indicated the action to be performed on the cup. The action was either to ‘lift’ or to ‘turn’ the cup (50% of the trials were allocated for each instruction, respectively).

A green horizontal line on the cup specified the initial hand posture to be used. If the line was at the top, participants had to grasp the cup using a supinated wrist posture, with their thumb facing ‘up’. If the line was placed at the bottom, participants were instructed to grasp the cup using a pronated wrist posture, with their thumb facing ‘down’. In each case, participants were asked to align their thumb and forefinger with the line. These grasps were made independent of the cup orientation. Hence in 50% of the trials, the initial wrist posture was congruent with the cup orientation (if the line was on the same side as the open end of the cup, such that the cup was grasped from its ‘lip’), and this was the case whether the cup was in its upright position (in which case the line was on the side of the open end of the cup and the grip was supinated) or upside down (in which case the grip was pronated).

The action, either to lift or turn the cup, determined the final end posture, which was again either pronated or supinated depending on the initial grip instruction and the action.

When the action was completed, participants returned their hand to the resting position, which led to the closure of the PLATO spectacles. Performance of this task was under full direct vision and the spectacles only closed after completion of the action and upon return to the resting position. They, therefore, remained open for the action for an average of 4 s (±0.5 s). Participants were asked to complete their action as quickly and as accurately as possible.

The experiment was programmed on Matlab 2014b using Psychtoolbox version 3.0, triggered from a windows PC. The experimenter recorded errors or adjustments in the grasp position, and changed the cup condition for the next trial. The movements performed on each individual trial were video-recorded and reviewed off-line, to assess and categorise any errors made by the participant, qualitatively. Of note, video recording in this task was not set-up to measure kinematic aspects of movement accuracy.

The video recordings led to the grouping of error trials into the following categories: (1) inappropriate grasps (they used a pronated wrist posture instead of a supinated one, instructed by the green line, or vice-versa) (2) wrong action (they lifted instead of turned or vice-versa), (3) adjustment in grasp posture prior to contact with object (for example reaching with a supinated grasp, when the green line on the cup indicated they should have used a pronated one, changing this before contact with the object) and (4) equipment or experimenter fault invalidating a trial. Although these errors were documented by the experimenter with every trial, they were looked at post-hoc to ensure the error was appropriately characterised. The aim of this categorisation was to enable comparison of participants’ performance with patients (Rounis et al. in preparation), but it was not used for the purposes of this study. As example, video recording is provided as supplementary material [video1].

All trial types (grasp top or bottom of cup, lift or turn) were presented in a pseudorandom order in a total of 17 mini-blocks, to ensure all trial conditions were repeated the same number of times, for averaging. Each mini-block consisted of one trial from each of the eight trial conditions. The first set of mini blocks was always eliminated from analysis. The total number of trials per session was 136, of which the first 8 were discarded, so only 128 were analysed for each participant.

This arrangement allowed us to measure response times at two time points, reflecting movement preparation for the action performed in this task. The Initiation time measured the time at which the participant lifted their hand from the spacebar where it was resting to initiate an action towards the cup. The Movement duration was the time between the release of the spacebar and the lift of the object from the platform, measured by the trigger of buttons from the cedrus box on which the platform was positioned.

These timings were based on previous studies, where the same two time points were chosen to investigate how object-related familiarity effects and end-state comfort effects emerged over time (Bub and Masson 2010; Rosenbaum et al. 1992). In the study by Rosenbaum et al. (1992), ‘T1’ was the time participants took to leave their hand from a predefined starting position, and corresponds to our ‘initiation time’; whereas ‘T2’ was the time to reach for the bar and remove it from the response panel, with the aim to position it onto a target, which corresponds to ‘movement duration’. It was assumed that the former primarily reflected processing and early planning of the forthcoming reach, whereas T2 primarily reflected later planning and the bulk of movement execution.

#### Data analysis

Any errors recorded by the experimenter, as well as correct response latencies lower than 200 ms or longer than 2000 ms for initiation times were excluded as outliers. The lower and upper bounds chosen for these time points were set so that no more than [0.5%] of correct responses were excluded either due to being classified as a false start or as an unusually prolonged response (see Ulrich and Miller 1994; Bub and Masson 2010, for a similar approach).

We hypothesised that actions in which the initial grasp went to the part of the cup linked to the cup’s normal function—i.e. to its open end—would be faster than a grasp to the non-functional (closed) end of the cup. Indeed, orienting the hand such that the thumb is aligned to the cup’s open end should enable its use, for drinking. One would predict that participant would be more likely to choose a hand orientation appropriate for the object’s use, as demonstrated previously (Creem and Proffitt 2001). For an upright cup this would correspond to a supinated grasp, for a cup oriented down, a pronated grasp (Fig. 1). Conversely, actions that were directed to the closed (end section) of the cup, would not be compatible with its learnt functional use, and would be slower.

The correct responses for each time point (initiation, and movement durations) were submitted to separate analyses of variance (ANOVAs) with the factors of initial grasp preference (grasp position of the hand in relation to the open end of the cup: this was designated as ‘preferred’ if the hand was oriented to the open end of the cup, independent of whether the cup was upright or down, i.e. a preferred initial grasp would be supinated if the cup was up and pronated if the cup was down; and there would be no grasp preference if the object was grasped from a non-familiar position i.e. its closed end), action (which determined whether the task was to ‘lift’ or to ‘turn’ the cup) and the end state comfort of the hand after the action [end state comfortable (‘thumb up’) or not (‘thumb down’)]. They were analysed using IBM SPSS Statistic 22 for windows software (SPSS Inc., Chicago, IL). The type I error rate was set at 0.05 for the analyses reported here. Greenhouse–Geisser correction for degrees of freedom was used when assumption of sphericity was not met. The same analysis was carried out on the error trials which were converted to mean percent errors and is provided below.

### Results

There were 1.45% of total errors with 1.26% of errors in grasp orientation, and 0.19% of errors of action. The results of the error rate analyses are reported below.

Reaction times for initiation shorter than 200 ms were found in 0.00015% of the total number of trials and ones longer than 2000 ms were found in 0.0003% of all trials, and were classified as outliers. These numbers were too low to perform any further statistical analyses. Error trials and outlier trials were excluded from the initiation and movement duration analyses reported below.

#### Initiation times

The ANOVA measuring the effects of task conditions on initiation times revealed significant main effects for initial grasp preference [*F*(1,29) = 16.76, MSE = 1379.6, *η* ^2^ = 0.37, *p* < 0.0001], action [*F*(1,29) = 7.1, MSE = 1844.6, *η* ^2^ = 0.2, *p* = 0.012] and end state comfort [*F*(1,29) = 14.5, MSE = 728.1, *η* ^2^ = 0.334, *p* = 0.001]. Initiation times for grasps which were aligned to the open end of the cup (i.e. with a preferred ‘initial grasp’), pulled across lift and turn actions and end state comfort, were 20 ms shorter on average (SEM 3 ms; *t*_29_ = −4.1, *p* < 0.001). Similarly, initiation times for lift actions pulled across initial grasp preference and end state comfort were, on average, 14.8 ms shorter than turn action (SEM 0.1 ms, *t*_29_ = −2.7, *p* = 0.006). Finally, initiation times for actions which ended in a comfortable end posture (with their ‘thumb up’), pulled across initial grasp preference and action, were on average 13 ms (SEM 0.8 ms, *t*_29_ = −3.8, *p* < 0.001) shorter than ones which ended in a ‘thumb down’ posture.

There were significant two-way interactions between initial grasp preference and end state comfort [*F*(1,29) = 7.8, MSE = 587.8, *η* ^2^ = 0.22, *p* = 0.009], as well as between action and end state comfort [*F*(1,29) = 18.5, MSE = 1580.2, *η* ^2^ = 0.39, *p* < 0.0001]. No other interactions were reliable.

**Fig. 2:**
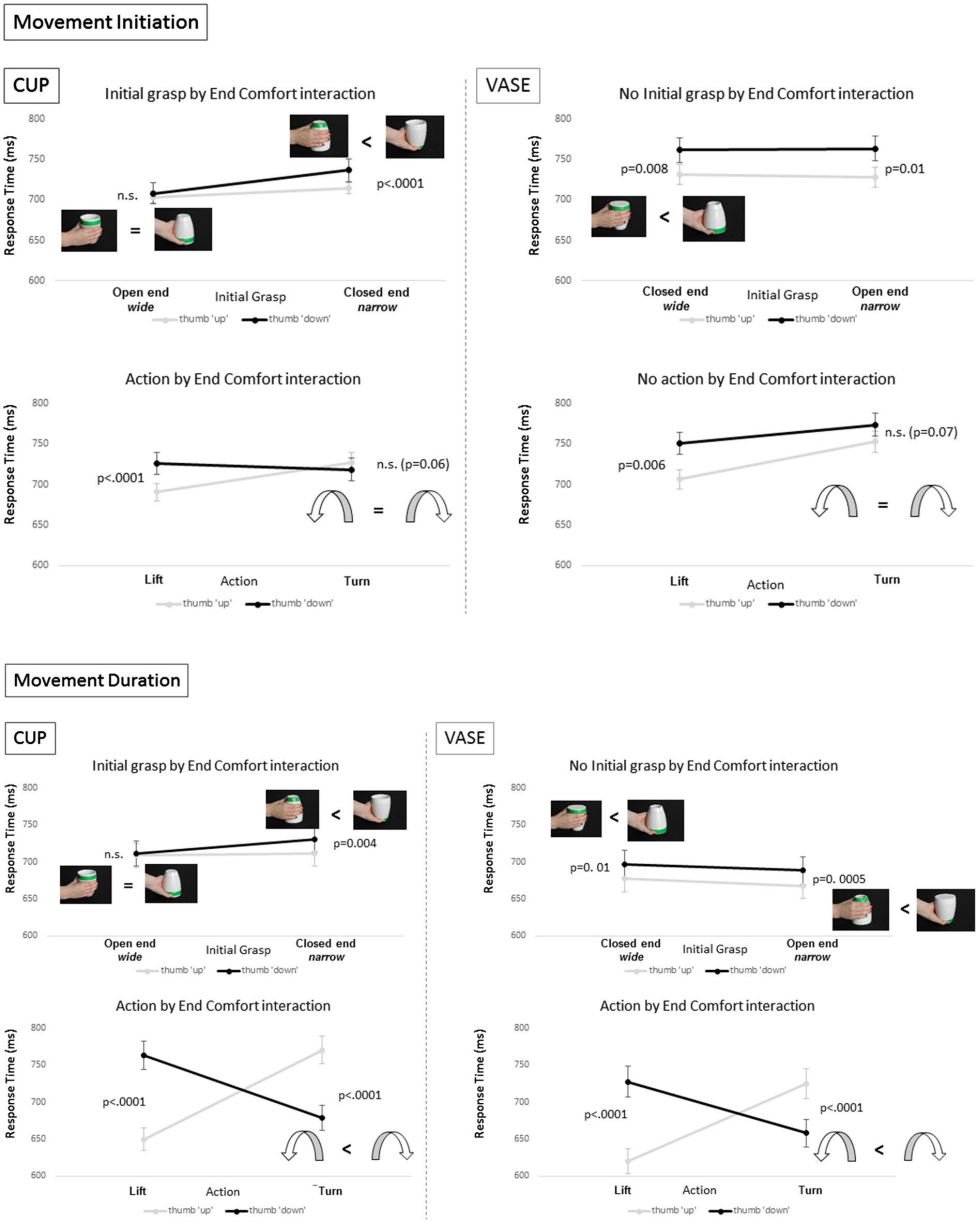
Initial grasp preference by end comfort and action by end comfort interactions for cups and vases from ANOVAs performed on reaction times at initiation and movement duration. In both *panels*, the abscissa represents the response time, whereas the ordinate represents the end-state position which was either comfortable (thumb ‘*up*’) or not (thumb ‘*down*’). The *top* graphs show the interaction between initial grasp preference and end-state comfort, which was significant for cups (*left*) and not for vases (*right*). This effect in the cup indicated that the object ‘affordance’ over-rides the movement plan to end comfortably, as there was no end-state comfort modulation of the initiation times or of the movement duration when the cup was grasped by its open end (i.e. grasping that side was ‘preferred’). The action by end-state comfort interaction indicated in the bottom graphs was present in both objects and reflected a possible cost of switching to start with an inverted grasp for a good end-state comfort position during turn actions. At movement initiation (*bottom* graph) there appeared to be a trend for an end-state comfort preference in the cup (of ending in a thumb ‘*up*’ position compared to thumb ‘*down*’), which was in the opposite direction for the vase. A direct comparison of this effect between the two objects (involving a 4-way ANOVA—see “Results”) was not significant

Post-hoc pairwise comparisons revealed that the interaction between initial grasp preference and end state comfort resulted from the fact that initiation times for actions in which the initial grasp was directed to the open end of the cup were 30 ms (SEM 2.5 ms) shorter than ones in which it was not (*t*_29_ = −2.8, *p* = 0.005). In these trial conditions, initiation times were not modulated by whether the end state posture was comfortable or not (*t*_29_ = −0.99, *p* = 0.33). Conversely initiation times in which the initial grasp was not directed to the functional end of the cup (i.e. it was directed to its closed end) were shorter if the action ended comfortably, in a ‘thumb up’ orientation and longer if they ended uncomfortably, in a ‘thumb down’ orientation (*t*_29_ = −4.6, *p* < 0.0001). These results are shown in Fig. 2.

The interaction between action and end state comfort showed that initiation times for lift actions were 35 ms (SEM 0.6 ms) shorter if they started and ended comfortably, in a ‘thumb up’, compared to a ‘thumb down’ position (*t*_29_ = −5.2, *p* < 0.0001). This was not the case for initiation times in turn actions in which there was a trend for them to be shorter (8.8 ms, SEM 0.9 ms) if they started with a supinated (comfortable) grasp and ended uncomfortably [i.e. with the ‘thumb down’ (*t*_29_ = 1.6, *p* = 0.06)] than vice-versa (start with pronated, uncomfortable grasp to end with a supinated, comfortable ‘thumb up’ orientation).

#### Movement duration

The main effects of initial grasp preference [*F*(1,29) = 7.8, MSE = 902.3, *η* ^2^ = 0.21, *p* = 0.009] and end state comfort [*F*(1,29) = 7.3, MSE = 1057.1, *η* ^2^ = 0.2, *p* = 0.01] were significant. The main effect of action was not [*F*(1,29) = 3.1, MSE = 6231.5, *η* ^2^ = 0.1, *p* = 0.088]. The interactions between initial grasp preference and end state comfort [*F*(1,29) = 5.7, MSE = 707.5, *η* ^2^ = 0.16, *p* = 0.02] and between action and end state comfort [*F*(1,29) = 102.4, MSE = 6177.2, *η* ^2^ = 0.78, *p* < 0.0001] were also significant. No other interactions were reliable.

The initial grasp preference by end state comfort interaction was similar to that found for initiation times. When the cup was grasped from its open end, movement durations were significantly shorter (10 ms, SEM 2.1 ms; *t*_29_ = −4.1, *p* = 0.00015) and they were not modulated by an end state comfort effect (*t*_29_ = −0.7, *p* = 0.48). In contrast, movement durations for actions in which the cup was grasped from its closed end were significantly modulated by the end comfort effect. The movement durations were on average 19.5 ms (SEM 1.04 ms) shorter if the actions ended comfortably (in a ‘thumb up’ position) than if they did not (*t*_29_ = −3.1, *p* = 0.004). The pattern of means for this condition are shown in Fig. 2.

The action by end state comfort interaction also followed the pattern for the initiation times. Lift actions which started and ended comfortably (in a thumb ‘up’ position) had shorter movement durations than actions which started and ended uncomfortably (in a thumb ‘down’ position) (*t*_29_ = −9.9, *p* < 0.0001). Conversely, movement durations for turn actions which started comfortably (in a thumb ‘up’ position) yet ended uncomfortably (in a ‘thumb down’ position) were 91 ms (SEM 6.6 ms) shorter than ones which started uncomfortably and ended comfortably (*t*_29_ = 8.8, *p* < 0.0001).

#### Percent error

The total number of errors recorded qualitatively in this experiment (see “Methods”) were converted to mean percent errors. These were entered into a 3-way ANOVA with the factors initial grasp preference, action and end-state comfort effect. This analysis revealed significant main effects of initial grasp preference [*F*(1,29) = 13.9, MSE = 0.002, *η* ^2^ = 0.33, *p* = 0.001], and a trend for a main effect of action [*F*(1,29) = 3.799, MSE = 3.6E^− 06^, *η* ^2^ = 0.11, *p* = 0.06]. There was no effect of end-state comfort on the mean percent errors [*F*(1,29) = 0.045, MSE = 8.05E^− 05^, *η* ^2^ = 0.002, *p* = 0.83]. Mean percent error rates were lowest when participants grasped the preferred side of the cup, i.e. its wide side, or open end (mean percent error rate difference: 1.3%, *t*_29_ = −3.7, *p* = 0.0008). Similarly, there was a trend for mean percent error rates to be lower for lift, compared to turn actions end (mean percent error rate difference: 0.5%, *t*_29_ = −1.9, *p* = 0.06). No other effects were significant. The initial grasp preference effect on error rate was generally consistent with that seen in the initiation times and movement durations, although the subtler interaction effects did not appear in the errors. There was nothing in the error data to suggest that any of the effects involving initial grasp preference, action or end-state comfort resulted from a speed-accuracy trade-off.

### Discussion

This experiment demonstrated the influence of an initial grasp preference determined by the object orientation: this grasp preference reflected properties of actions towards this specific object, namely a cup. Actions in which the instructed grasp was targeted to its open end, whether the cup was oriented in its upright position or down, were faster, even though the instructed actions—to ‘lift’ or to ‘turn’ the cup- were abstract in relation to its function. Similar results, of an initial grasp preference, have been reported when participants perform actions that are congruent with the orientation of an object, even if the action to be performed does not require use of that object (Tucker and Ellis 1998; Creem and Proffitt 2001; Bub and Masson 2010). Some authors have reported that these effects relate to object ‘affordance’ (discussed below).

In addition, we have shown an end state comfort effect (Rosenbaum et al. 1990, 1992). Actions in which the cup was grasped from its open end were faster and unaffected by the final end posture. In contrast, grasps made to the bottom, closed part of the cup, were initiated and completed more quickly when they resulted in a comfortable as compared to an uncomfortable end state. The results are consistent with an initial preference to select one’s grasp towards a learned target (e.g. grasping the open part of the cup) being dominant in movement planning and being still present during execution, and the end state comfort effects moderating performance primarily when there was not a strong effect of the former factor competing for the selection of the action. Hence in this case affordance over-rode movement planning. Unlike our prediction, which was that these effects would emerge at different time points during preparation and early components of execution of the action, our findings were present both in the initiation times and in movement duration.

A second finding was that the action interacted with the end state comfort. For lift actions, movement initiation times and durations were shorter when the action would start and end in a comfortable end state (with the thumb ‘up’). For turn actions, however, this effect was reversed—now actions ending in a poor end state comfort position were initiated more quickly than those ending in a good end state comfort. Turn actions which ended in a comfortable end state involved a hand inversion at the start, similar to a lift condition with a poor end state. Conversely, turn actions which ended with a poor end state involved a supinated grasp, similar to lift actions with a good end state. Inverting the wrist to grasp the cup at the start of the action may be difficult to programme, leading to slowing in the initiation times and movement durations. Importantly, these effects were independent of whether the grasp was made to the open or closed part of the cup.

The results obtained from this experiment raise the question of whether the observation of a preferred grasp towards the open end of the cup related to its function (drinking or filling it) or simply to its shape. The cup used in this experiment had slightly wider dimensions at its open end than at its closed end. In other words, did we observe this effect because it was biomechanically ‘easier’ to grasp the wider end of the cup, which happened to be the open end, or was this observation relating to a learnt behaviour, that the open end of the cup is the usual place to grasp it to perform meaningful actions such as to drink from it?

To address this, we designed a second experiment using a vase, which was matched in terms of its dimensions but for which the function (to ‘fill’) could only be achieved on the opposite side of the object. Hence this time, the open end was on the narrow part of the object and the closed end was the wider part, which formed its base.

## Experiment 2: reaching to grasp a vase

### Participants

A new set of thirty right-handed healthy participants [mean age was 27.3 years, *M* = 14:*F* = 16], who were naïve to the previous study, were recruited from advertisements in the Department of Experimental Psychology and provided informed consent in accordance with the University of Oxford ethics committee.

### Materials and methods

The trial sequence and experimental conditions were the same as in Experiment 1, except that participants performed the task using a ‘vase’. This was created out of a cup by changing the orientation of its open end to be on the side of its narrow base. The wide-base was covered with silicone (and had a hard texture). Hence the vase retained identical dimensions (in terms of shape, and weight) to the cup but differed in that the open (and therefore the ‘functional’) side of the object was narrow rather than wide. The reason for doing this control was to investigate whether the effect of the initial grasp preference related to the object shape, leading to a preference for a wide as opposed to a narrow grasp.

Of note the centre of gravity for the two objects were opposite to one another with relation to the open side of the object, but in the same location when comparing them to the narrow side of the object (centre of mass from narrow end of vase, which in this case was its open end, was 7.6 cm).

**Fig. 3:**
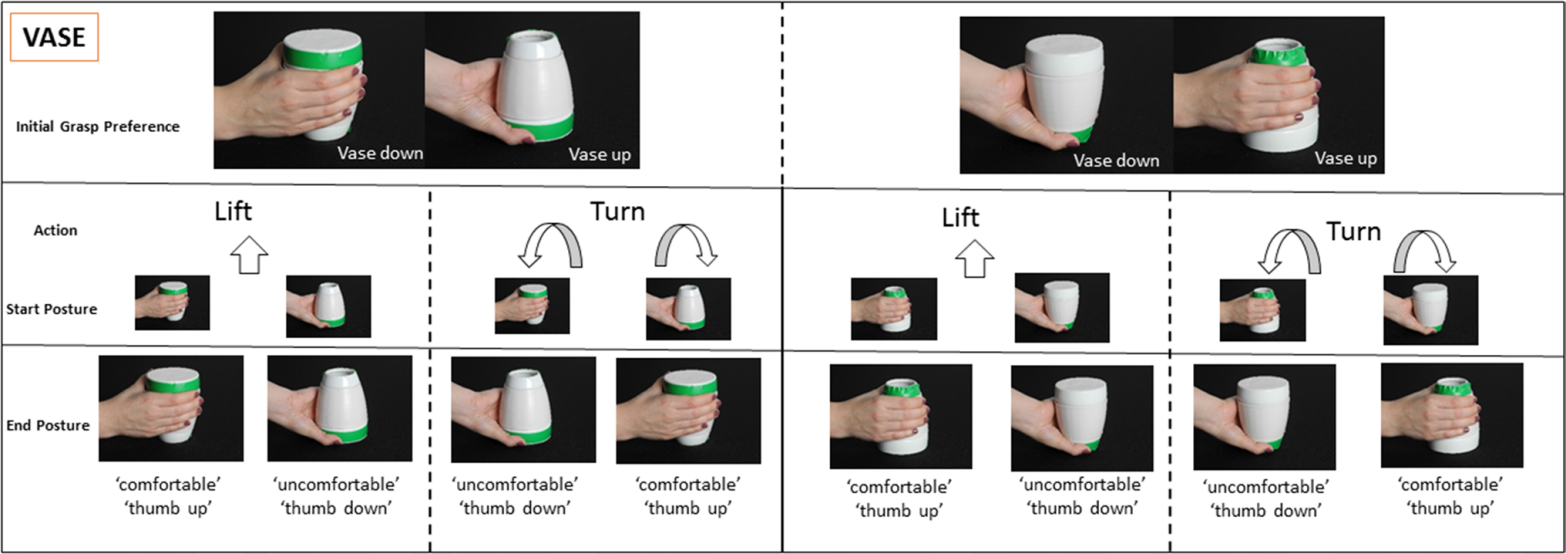
Experimental Conditions for the vase: this time the initial grasp preference corresponded to grasping the wide side of the object (which was the closed end). The action and end comfort state conditions were the same as in the cups

In this task, and for the purposes of comparison with the cup, we made the assumption that actions to the wide base of the vase, which was its closed end, were the preferred side by which the object was being grasped. The wide side was identified as demonstrating an initial grasp preference in the cup. If the reason why it might have been ‘preferred’ in the previous experiment was due to the shape of the object, then we predicted that the effects of ‘initial grasp preference’ identified in the cup would also be present for the vase. If this was not the case, then we could assume that object shape was not the only reason driving that ‘initial grasp preference’ in the cup, and that other reasons, such as knowledge about its function, might play a role. The object (vase) and experimental conditions are shown in Fig. 3.

Participants completed one session of 17 blocks of trials, each comprising 136 trials, of which the first 8 trials were discarded, leaving 128 trials for analysis for each session.

Movement initiation and duration times were entered into separate analyses of variance comprising the same factors as in the cup. The *initial grasp preference* was defined as the grasp orientation that was compatible with the wider side (in this case the closed end) of the object. We hypothesised that the findings reported in Experiment 1 might have related to a preference for grasping the wider side of the object because it was physically ‘easier’. According to this hypothesis, this grasp would be preferred both for the cup and the vase, making the initiation and movement duration faster in these conditions.

### Data analysis

The correct responses for each time point (initiation, movement duration) were submitted to separate analyses of variance (ANOVAs) for each object (the cup, and the vase) with the factors of initial grasp preference (grasp orientation of the hand in relation to the wide side of the vase), action (which determined whether the task was to ‘lift’ or to ‘turn’ the cup) and the end state comfort of the hand after the action (comfortable if the end posture was in a ‘thumb up’ configuration, uncomfortable if it was in a ‘thumb down’ configuration).

A second, between-group analysis was performed to formally compare the results of the cup and the vase. Initiation times and movement durations were entered into separate repeated measured ANOVAs, respectively, with the between-group factor of object (cup versus vase), and the within-group factors of initial grasp preference, action and end state comfort, as before.

Repeated measures ANOVAs were analysed using IBM SPSS Statistic 22 for windows software (SPSS Inc., Chicago, IL). The type I error rate was set at 0.05 for the analyses reported here. Greenhouse–Geisser correction for degrees of freedom was used when assumption of sphericity was not met. Interaction effects were evaluated with paired *t* tests (*p* ≤ 0.05).

### Results

There were 2.47% of errors in total with 1.65% errors in grasp orientation (adjusting hand postures prior to contact with the object, as described in experiment 1) and 0.82% errors in action performing a lift instead of a turn and vice versa). The results of the error rate analyses are reported below.

Reaction times for initiation times shorter than 200 ms were found on 0.007% of the total number of trials and ones longer than 2000 ms were found on 0.004% of all trials and were classified as outliers. The number of outliers was too low to perform any further statistical analyses. Error trials and outliers were excluded from the time analyses reported below.

### Initiation times

There were significant main effects of action [*F*(1,29) = 7.53, MSE = 9483.1, *η* ^2^ = 0.21, *p* = 0.01] and end state comfort [*F*(1,29) = 8.9, MSE = 7152.6, *η* ^2^ = 0.24, *p* = 0.006]. Unlike Experiment 1, the main effect of initial grasp preference was not significant [*F*(1,29) = 0.017, MSE = 1345.9, *η* ^2^ = 0.001, *p* = 0.9]. Initiation times to lift the vase were on average 34.5 ms (SEM 10 ms) shorter than to turn it (*t*_29_ = −2.7, *p* = 0.005). Actions which ended comfortably (in a ‘thumb up’ position) were initiated 32.7 ms (SEM 2.7 ms) faster than ones which ended uncomfortably (in a ‘thumb down’ position) (*t*_29_ = −2.9, *p* = 0.003). There was no significant difference in initiation times between actions that were targeted to the wide side of the vase (mean 746.4 ms SEM 39.5 ms) compared to ones that were targeted to its narrow side (‘open end’) (mean 745. 8 ms, SEM 40.4 ms) (*t*_29_ = 0.13, *p* = 0.5).

Moreover, no two- or three-way interactions were significant. Notably, action by end state comfort [*F*(1,29) = 2.45, MSE = 3213.4, *η*_*p*_^2^ = 0.078, *p* = 0.13] and initial grasp preference by end state comfort [*F*(1,29) = 0.311, MSE = 1405.9, *η*_*p*_^2^ = 0.011, *p* = 0.58] interactions were not significant.

### Movement durations

Similar analyses performed on the movement durations revealed significant main effects of action [*F*(1,29) = 16.52, MSE = 1134.8, *η*_*p*_^2^ = 0.36, *p* < 0.0001] and end state comfort [*F*(1,29) = 16. 2, MSE = 1134.8, *η*_*p*_^2^ = 0.36, *p* < 0.0001]. Movement durations when the action was to lift were 17.7 ms (SEM 0.9 ms) shorter than when the action was to turn (*t*_29_ = −4.1, *p* = 0.0003). Movement durations for actions which ended comfortably (in a ‘thumb up’ position) were 20 ms (SEM 1.7 ms) shorter than ones which ended uncomfortably (*t*_29_ = −4.0, *p* = 0.0002). The main effect of initial grasp preference was not significant for the vase [*F*(1,29) = 2.6, MSE = 1525.9, *η*_*p*_^2^ = 0.08, *p* = 0.12].

There was a significant two-way interaction of action by end comfort [*F*(1,29) = 76.4, MSE = 6036.6, *η*_*p*_^2^ = 0.72, *p* < 0.0001]. As with the cup in Experiment 1, lift actions which started and ended comfortably (in a thumb ‘up’ position; mean 620 ms, SEM 37.8 ms) had shorter movement durations than actions which started and ended uncomfortably (in a thumb ‘down’ position; mean 727.8 ms, SEM 45 ms) (*t*_29_ = −7.9, *p* < 0.0001). Conversely, movement durations for turn actions which started comfortably (in a thumb ‘up’ position) yet ended uncomfortably (in a ‘thumb down’ position) were 67 ms (SEM 3.9 ms) shorter than ones which started uncomfortably and ended comfortably (*t*_29_ = 8.2, *p* < 0.0001). There were no further interactions.

#### Percent error

As in Experiment 1, the total number of errors were converted to mean percent errors, which were entered into a 3-way ANOVA with the same factors as in the initiation and movement duration times, namely initial grasp preference, action and end-state comfort effect. None of the main effects or interactions was significant in the vase. As with the cup, there was no evidence of a speed-accuracy trade-off in the mean percent errors.

### Comparison between the cup and the vase

To compare the effects of the cup and the vase directly, we added object (cup versus vase) as a between-subject factor in a further two separate ANOVAs for the initiation times, and movement durations respectively. Of interest were the interaction effects between object and the other factors. These are reported below.

### Initiation times

The main effects of initial grasp preference [*F*(1,29) = 7.5, MSE = 1362.8, *η*_*p*_^2^ = 0.2, *p* = 0.01], action [*F*(1,29) = 12.9, MSE = 5663.8, *η*_*p*_^2^ = 0.18, *p* = 0.001] and end state comfort [*F*(1,29) = 16.1, MSE = 3940.4, *η*_*p*_^2^ = 0.22, *p* < 0.0001] were significant. Initiation times were, on average, 30.1 ms (SEM 2.2 ms) shorter for grasps targeted to the wide side of the object (*t*_29_ = −2.7, *p* = 0.005); they were 24.6 ms (SEM 5 ms) shorter for lift, compared to turn actions (*t*_29_ = −3.2, *p* = 0.002); and they were 23 ms (SEM 4.7 ms) shorter for actions that would end comfortably (in a thumb ‘up’ position) compared to ones ending in an uncomfortable, thumb ‘down’, position (*t*_29_ = −3.6, *p* = 0.0006). The main effect of object was not significant [*F*(1,29) = 0.31, MSE = 1567.2, *η*_*p*_^2^ = 0.01, *p* = 0.58].

However, there was an object-by-initial grasp preference interaction [*F*(1,29) = 9.6, MSE = 1272.2, *η*_*p*_^2^ = 0.14, *p* = 0.004], reflecting that the initial grasp preference effect was much stronger for the cup (*t*_29_ = −4.1, *p* = 0.00015) than for the vase (*t*_29_ = 0.13, *p* = 0.45). There were no other significant 2-way interactions with the factor ‘object’. The two-way initial grasp preference by end comfort [*F*(1,29) = 1.1, MSE = 996.9, *η*_*p*_^2^ = 0.04, *p* = 0.3] and action by end comfort [*F*(1,29) = 0.71, MSE = 1896.9, *η*_*p*_^2^ = 0.02, *p* = 0.4] interactions were also not significant.

Nevertheless, there was a trend for a three-way object-by-initial grasp preference by end comfort interaction [*F*(1,29) = 3.95, MSE = 2936.9, *η*_*p*_^2^ = 0.12, *p* = 0.052], which reflected that there was a much stronger initial grasp preference by end state comfort interaction for the cup [*F*(1,29) = 7.835, MSE = 364.1, *η*_*p*_^2^ = 0.293, *p* = 0.009] than for the vase [*F*(1,29) = 0.311, MSE = 703, *η*_*p*_^2^ = 0.011, *p* = 0.6], when this interaction was further decomposed into two separate two-way ANOVAs for each object. These results are detailed in Experiment 1. There were no other reliable three-way interactions.

### Movement durations

The main effects of action [*F*(1,29) = 10.4, MSE = 3683.1, *η*_*p*_^2^ = 0.15, *p* = 0.002] and end state comfort [*F*(1,29) = 1.8, MSE = 1279.3, *η*_*p*_^2^ = 0.29, *p* < 0.0001] were significant. Movement durations for lift actions were on average 17.8 ms (SEM 1.5 ms) shorter than for turn actions (*t*_29_ = −3.2, *p* = 0.0015); movement durations for actions which ended in a comfortable posture (thumb ‘up’) were on average 15.7 ms (SEM 1.1 ms) shorter than ones which ended with an uncomfortable posture (thumb ‘down’).

The main effects of object [*F*(1,29) = 0.3, MSE = 41996.9, *η*_*p*_^2^ = 0.01, *p* = 0.6] and initial grasp preference [*F*(1,29) = 0.053, MSE = 1413.65, *η*_*p*_^2^ = 0.002, *p* = 0.82] were not significant for movement durations.

However, there was a significant object by initial grasp preference interaction [*F*(1,29) = 12.2, MSE = 992.2, *η*_*p*_^2^ = 0.3, *p* = 0.002]. A post-hoc two-way ANOVA with the factors object (cup versus vase) and initial grasp preference (to the wide versus to the narrow side), revealed no significant main effects (object [*F*(1,29) = 0.29, MSE = 112992.2, *η*_*p*_^2^ = 0.01, *p* = 0.595]; initial grasp preference (for wide side) [*F*(1,29) = 0.053, MSE = 353.4, *η*_*p*_^2^ = 0.002, *p* = 0.82]) but confirmed an interaction between the two [*F*(1,29) = 12.2, MSE = 248.1, *η*_*p*_^2^ = 0.3, *p* = 0.002]. Post-hoc *t* tests revealed this was due to a significant effect of initial grasp preference in the cup. As described in the results’ section in Experiment 1, movement durations were shorter when grasping the wide (open) side of the cup, deemed to be its functional side (*t*_29_ = −2.8, *p* = 0.005). This was not the case in the vase (*t*_29_ = 1.6, *p* = 0.11).

Finally, there was a significant two-way action by end comfort interaction [*F*(1,29) = 263.8, MSE = 4120, *η*_*p*_^2^ = 0.9, *p* < 0.0001], depicted in Fig. 2. Movement durations for lift actions were on average 39.8 ms (SEM 5 ms) shorter when the start and end hand postures were comfortable (in a thumb ‘up’ position), compared to when these postures were uncomfortable (in a thumb ‘down’ position; *t*_29_ = −15.4, *p* < 0.0001). In turn actions, movement durations for grasps which started in a thumb ‘up’ and ended in a thumb ‘down’ position were executed faster than vice versa (*t*_29_ = 13.4, *p* < 0.0001), likely reflecting a cost of switching to start with an inverted grasp for a good end-state comfort position. No further two- or three-way interactions were identified.

#### Percent error

The mean percent errors from Experiment 1 and 2, were entered into a 4-way ANOVA with the factors object, initial grasp preference, action and end-state comfort effect. There were no significant main effects or interactions identified in this analysis.

### Discussion

The aim of Experiment 2 was to explore whether an ‘initial grasp’ preference found in Experiment 1, related to the physical or to the functional properties of the object (the wide side of cup corresponding to the side it is usually grasped from). Here, we used a vase with the same physical dimensions as the cup but with its opening at the narrow rather than the wide side.

The initial grasp preference and its interaction with the end state comfort effect, which were identified with the cup, were not replicated for the vase.

Interestingly, we found effects of the object type on initial grasp preference, in all phases of movement planning in this task. There was a three-way interaction of initial grasp preference by object, by end comfort state at initiation times, whereas movement durations revealed a simple two-way object by initial grasp preference interaction.

The former, three-way interaction, suggests that the interaction of grasp and end state comfort found for the cup reflected an ‘over-learned’ response to the open section of the cup. The strong activation of this familiar grasp was sufficient in the cup to overrule effects of end state comfort. For the vase grasping the wide or narrow part of the object did not impact on the end state comfort effect, meaning that this effect persisted in leading to faster preparation times when the action ended comfortably than when it did not. The latter, two-way, interaction, suggests that the influence of an ‘initial grasp preference’ to the wide (and open) side of the cup, was not present in the vase, such that when implementing the action, it was not the physical property of the object (its wide side) that led to faster movement durations in the cup, but rather the ‘object property’ relating to its open end representing where it is to be used.

In addition, there were effects of action and end state comfort on both objects. Initiation times and movement durations for lift actions were shorter when the grasp postures at the start and at the end were comfortable (i.e. in a ‘thumb up’ position). Conversely, movement durations for turn actions were shorter when the grasp postures at the start were comfortable (in a thumb ‘up’ position) even though the action would result in an uncomfortable end state (with the thumb ‘down’). This suggests that for turn actions there appeared to be a ‘start’ as opposed to an ‘end’ state comfort effect, which occurred during the reaching phase of the action. This likely reflected the fact that actions which ended comfortably involved a wrist inversion ‘in-flight’ whilst reaching to grasp the object which took longer when measuring the reaching-to-grasp-part of these actions.

Taken together these findings suggest a dynamic modulation of end comfort and initial grasp which was both contextual and object-specific.

## General discussion

Our studies explored factors influencing motor planning when grasping an object. One factor is the end state comfort effect, originally reported by Rosenbaum and colleagues (1990, 1992, 1996, 2012, 2014). This effect describes the choice people make when grasping an object to start their action uncomfortably, so that they reach a comfortable posture at the end. We measured this effect on movement preparation, which was faster in our task for actions ending comfortably compared to ones which ended uncomfortably. We used a task in which grasps to interact with a familiar object, namely a cup, were specified in advance. Our aim was to investigate the relations between the end state comfort effect and the familiarity of the grasp to the functional part of an object.

Several factors were identified to influence these effects during motor preparation in our study. First, we observed that the action ‘goal’, provided by the instruction to ‘lift’ or to ‘turn’ modulated the end-state comfort effect. Whereas lift actions were prepared faster if they started and ended comfortably than if they did not, there was less modulation (or even a reverse modulation during reaching) of end state comfort on the speed of preparation for turn actions. This reflected the preparation times we measured, which involved the reaching part of the action. If the action was to turn, aiming to achieve a comfortable end state required the wrist to be pronated at the start (an uncomfortable position), which led to longer preparation times, even though the action would lead to a preferred (supinated) wrist position at the end.

Second, we identified an ‘initial’ grasp preference, which not only influenced the speed of motor preparation but also modulated the end state comfort effect on that speed. The initial grasp preference related to grasping the object from its ‘natural’ side. In the case of a cup, grasping it from its open end led to faster preparation times, overall. Moreover, this effect had an influence on end state comfort, which was reduced or absent in trials where participants grasped this ‘preferred’ side. Hence, the initial grasp preference over-ruled the end state comfort effect in these cases.

In our experiment, the cup shape looked like an inverted cone. This meant that the open end (or functional/familiar part of the object) was also wider in size. Our finding of an ‘initial grasp preference’, in which movement preparation to lift or turn the cup was performed faster, could either be related to the size of the open end of the cup being ‘wider’, or to familiarity of performing actions toward the functional part of the object.

We carried out a second experiment to investigate this further. Here, we asked whether the initial grasp preference was determined by the physical properties of the object, instead of knowledge about its function. Using a vase, with the same dimensions as the cup but the open end located on its opposite side, we found that this initial grasp preference effect was not significantly affecting the speed of movement preparation (i.e. movement preparation times were no faster when the vase was grasped from its wide, as compared to its narrow (and open) sides). We conclude that this initial grasp preference effect was most likely driven by a familiarity effect, of grasping the cup from its open end. This was not present for the vase; despite the fact the object had the same physical dimensions as the cup. We suggest that the vase does not elicit such a strong overlearned grasp response as the cup.

A previous study identified that even a simple action to an object can elicit grasping it from its functional side (Creem and Proffitt 2001). When participants in this study were asked to grasp everyday objects during dual performance of a visuospatial versus a semantic task, they typically grasped familiar objects from their functional side unless the cognitive demands of the semantic task were too high (Creem and Proffitt 2001). Taken together, these results would suggest that this ‘initial grasp preference’ effect, also identified in our study, likely represents semantic knowledge about the object rather than a simple visuospatial congruency effect.

### Factors influencing planning of actions to objects

In 1979, Gibson introduced the concept of ‘affordances’ which he described in terms of the relationship between the physical properties of an object, a functional goal, and the physical properties of an actor. An action is afforded if the physical properties of an object can be linked to a functional goal and the actor’s motor capabilities. We would argue that the initial grasp preference effect observed in our study relates to this concept of affordances.

A number of behavioural tasks have attempted to explore how affordances modulate human performance. Further to a seminal experiment, Tucker and Ellis (1998) proposed that visuomotor relations between objects and actions activate a motor response for object-use automatically, even when the motor response is not required by the task. Subsequent studies have shown that similar compatibility effects could be elicited for different aspects of objects (e.g. their size or orientation) (Ellis and Tucker 2000; Symes et al. 2008). The effects identified in these studies have led to debates about whether they represent a simple visuospatial compatibility effects or whether objects/object features trigger movement representations targeted to their use (Bub and Masson 2010; Kornblum and Lee 1995; Phillips and Ward 2002; Wilf et al. 2013).

There is evidence from the neuropsychology literature for the latter, that is, objects in the environment may, in fact, elicit object-specific actions (Riddoch et al. 1998; McBride et al. 2013). In one study, a patient with corticobasal degeneration and alien-limb syndrome was asked to reach and grasp a cup using the hand that was on the same side of the table as the cup, regardless of which way its handle was oriented (Riddoch et al. 1998). The patient performed the task correctly when the cup’s handle was on the same side as the hand she was instructed to use. However, if the handle was on the opposite side, there were ‘interference’ errors: in this case, the patient was unable to inhibit the action of grasping the cup with the opposite hand, the action cued by the orientation of the cup’s handle in relation to the patient’s preferred hand. These errors were not present when she was asked to point to the object or responded to lights instead of cups, suggesting that for this patient, the simple observation of a graspable object might be sufficient to elicit the associated motor plan for interacting with that object, and was movement-specific.

Further evidence in support of the theory that affordances automatically trigger movement representations, comes from neurophysiological studies (Cisek and Kalaska 2005; Cisek 2007; Fagg and Arbib 1998). Cisek and Kalaska (2005) showed that recording activity in the dorsal premotor cortex in non-human primates, whilst they prepare to reach for one of two targets (with either arms), identified both movements being represented simultaneously, prior to choosing the target to be reached. A previous study had shown that movement representations for both arms could be elicited in this way, even when the animals were constrained to respond with only one arm by immobilising the other one, hypothesising a role for affordances in eliciting abstract levels of movement planning that could even be ‘effector independent’ (Cisek et al. 2003).

The ‘affordance’ effects we observed in this study extend previous findings, reported above. In addition to identifying an initial grasp preference with this task, we demonstrated that this effect was ‘object-specific’. It related to the cup, and was not identified in the vase, which had the same dimensions. This effect also influenced goal-directed planning by affecting the end-state comfort effect. The end state comfort effect, reflecting biomechanical properties of the action, was reduced in the cup, yet remained unchanged for the vase, independent of which side was being grasped.

These results suggest that the initial grasp preference likely reflected a preference in carrying out an action to the object that was over-learnt, due to familiarity with that object. However, we cannot claim, based on our results, the physical properties of the object had no influence at all, as indeed, both previous literature and comparative analyses of these effects on movement preparation suggested they were (there was a main effect of initial grasp preference at movement initiation for both objects, in the analyses comparing the two objects). Nevertheless, it is unlikely that our results could simply be explained by the physical properties of the cup. If the main driver of our effects related to physical properties of the object, we would have expected a reverse ‘initial grasp preference’ effect for vases (e.g. faster actions to the narrow yet functional part rather than the wide part of the vase), and a different pattern of interactions, which were not observed here.

It is currently not known how perceptual features of objects are integrated with semantic knowledge of their use, as is suggested in this study. The dual streams model for visual processing distinguishes between two brain networks, that likely underlie these respective processes. These include a dorsal visuomotor stream that uses perceptual features (such as the object’s size and shape) to specify goal-directed actions, and a ventral stream that is geared towards identification and recognition of objects (Goodale and Milner 1992). A previous neuroimaging study (Mruczek et al. 2013) suggests that the visual-motor and object-semantic processes are mediated by different neural substrates: the former is associated with the anterior portion of the intraparietal sulcus while the latter is associated with the posterior portion. However, the network of brain regions representing affordances likely incorporates semantic inputs from ventral stream structures to guide action representations in the dorsal stream (van Polanen and Davare 2015). Previous neuroimaging studies investigating the neural networks underlying object affordances, implicate areas within the dorsal stream, in interactions with anterior parietal cortex when actions must be selected on the basis of arbitrary cues, (Chao and Martin 2000; Grezes et al. 2003). The role of semantic representations on effects of affordances from familiar objects (which are more complex) remains unknown.

Interestingly, recent literature on the neural correlates of end state comfort effects, have identified a role for a ventral stream structure, namely the extrastriate body area (EBA) in representing postural configuration during actions that require anticipation of future states (Zimmermann et al. 2012). Further investigations of the role of this area using transcranial magnetic stimulation (TMS) suggests that it provides the dorsal visuomotor stream with a representation of a desired goal state that enables the dorsal visuomotor stream to specify an appropriate motor plan (Zimmermann et al. 2016). It is possible that semantic inputs are used to guide movement planning to familiar objects in a similar way. Further studies using neuroimaging and / or neurophysiological methods (e.g. ERP/MEG data or even TMS) could investigate this possibility and allow a better understanding of how this is achieved, neuronally.

### A unifying account of the effects of affordance and end state comfort

In this study, we hypothesised we would identify effects of an ‘initial grasp preference’, and ‘end comfort state’, which would emerge dynamically during movement preparation. We used two time points to measure how these influences on motor preparation emerged over time. The initiation time measured the time the participants’ hand lifted off from a resting position to initiate the action. Traditionally this is felt to measure processes involved in deciding which action to perform and how to implement it (Welford 1968; Wong et al. 2015). The movement duration measured the transport phase of the action, as participants reached to grasp the object. It was used to reflect preparation of effector-based movement implementation (Rosenbaum et al. 1992; Cisek et al. 2003; Bub and Masson 2010). The differences relating to perceptual effects of the object on end state comfort planning were identified at movement initiation and persisted in the reach-to-grasp phase measured by movement durations. As mentioned in the previous section the exact mechanisms of how these processes are integrated over time will require further investigations.

We propose an interpretation of our results similar to that suggested in a study by Chainay and Humphreys (2002). They proposed that action selection arises dynamically by a process of convergence incorporating perceptual information via a direct visual route with knowledge about an action from an ‘indirect’ semantic route. Using an ‘energy minimisation’ network (Hopfield 1982), motor responses (ie movements) are likely determined by convergent activation from these separate routes. This convergent activation ‘pushes’ the network into ‘stable states’ that represent the learned output for a given stimulus. An example of direct visual input triggering an action is the sight of a button triggering the action to press it (i.e. an ‘affordance’), or in our study the sight of the open end of the cup triggering the action to grasp it from its open side (or its ‘lip’). This stable state acts as an attractor which dynamically pulls any initial activation supplied by the visual stimulus toward it (e.g. open side of cup), along with any other incoming inputs (e.g. it is upright) into a ‘basin of attraction’ (e.g. knowledge of what one does with a ‘cup’). According to this, semantic information determines the appropriate category of action, whilst the direct perceptual input helps to determine the optimal parameters for the motor programme (e.g. the grip aperture).

This framework has been used to explain neuropsychological deficits such as limb apraxia, a syndrome causing the inability to conceptualise or implement appropriate, skilful, actions, despite intact motor and sensory apparatus, and comprehension (Rothi et al. 1991). Patients with apraxia who have deficits in object-use, often show paradoxical improvement with the object ‘at hand’. The model proposed by Chainay and Humphreys (2002) helps provide a mechanism by which this can occur. In patients with ideational apraxia showing an ‘object at hand’ benefit, this is likely due to perceptual information ‘driving’ actions (as attractors) by pulling them into basins of attraction that enable the appropriate use of that object in spite of limited access to semantic knowledge. It has also been used to explain blocking effects in modality-specific apraxias, with examples of apraxic patients being able to carry out an action with one hand but not the other if they are provided with visual information, and vice versa if they are provided with semantic or verbal information (Riddoch et al. 1989). In a similar vein, this framework can explain ‘blocking’ effects of one component of perceptual information onto another (Chainay and Humphreys 2002). More recent evidence is emerging from neurophysiology literature in non-human primates, suggesting that neuronal activity in the primary motor cortex during motor preparation recorded a little prior to the onset of a reaching movement, has dynamic properties which can be modelled using state space models akin to the neural networks suggested above (Churchland et al. 2012).

In our study, we propose that there are different ‘activation spaces’ for the various components parts of a movement to the cup: at action programming (indexed by the initiation time), activation is ‘pushed’ into a given basin of attraction based on constraining variables including the position of the hand in relation to the part of the object linked to the usual functional goal, the position of the object and factors such as the end state comfort which represent the outcome of the action in our task. For the initial stages of action selection, we suggest that there are strong effects of hand and object position, so that afforded actions are rapidly selected, ‘blocking’ effects of end state comfort planning. However, when the hand and object locations are incongruent, then other variables, such as the end state comfort, appear to modulate performance.

In conclusion, we were able to demonstrate effects of an initial grasp preference, influenced by overlearnt patterns of object use and likely to represent ‘affordances’ as defined by Gibson (1979) on planning actions to an object, and end state comfort effect, indicating that habitual actions to an object biased planning to favour actions which are normally done. While previous studies demonstrate these effects separately, we provide novel behavioural evidence that they interact with each other during the course of action planning. We propose the integration of semantic and perceptual information to achieve this. Further work in this field will enable us to clarify the dynamic interaction of these factors, which determine action selection and planning, and their modulation over time.

## Electronic supplementary material

Below is the link to the electronic supplementary material.

Supplementary material 1 (MOV 470 KB)
